# Reentry of voluntary blood donors in Chongqing, China (2022–2024): a retrospective single-center analysis

**DOI:** 10.3389/fmed.2026.1780036

**Published:** 2026-03-20

**Authors:** Weifei Qin, Tingting Hu, Yubi Gan, Yunbo Tian, Xiaobing Zhu

**Affiliations:** Chongqing Blood Center, Chongqing, China

**Keywords:** donor reentry, single-reagent reactivity, blood donor, blood screening, requalification

## Abstract

This study presents a retrospective analysis of the reentry process for voluntary blood donors in Chongqing, China, from 2022 to 2024, focusing on donor profiles, requalification outcomes, and factors influencing successful return to donation. Donor reentry allows individuals who were temporarily deferred due to reactive screening results for transfusion-transmissible pathogens to requalify as eligible donors, thereby helping to reduce unnecessary donor loss and alleviate donor distress. However, the implementation and effectiveness of such programs vary considerably across regions in China. In this analysis, 808 donors who applied for reentry at the Chongqing Blood Center were included. Reentry testing followed a structured protocol consisting of dual-ELISA serology, individual nucleic acid testing (NAT), and supplementary chemiluminescence assays. Categorical variables were analyzed using the chi-square test in SPSS 27.0. The overall reentry success rate was 60.52%, with the highest rate observed among HIV-reactive donors (68.85%) and the lowest among HBV-reactive donors (40.65%). Higher educational attainment was significantly associated with reentry success, while sex, ABO blood type, marital status, and occupation showed no significant correlation. Of the successfully re-entered donors, 49.08% returned to donate at least once, although most donated only once or twice subsequently. Disqualifications occurred in 13.75% of returning donors, primarily due to reactivity for HIV, HBV, or TP. The findings indicate that a well-defined reentry protocol can effectively reintegrate a substantial proportion of single-reagent-reactive donors into the eligible donor pool. Requalification outcomes are influenced by pathogen type and donor education level, underscoring the need for pathogen-specific testing strategies and tailored donor communication. Sustained donor engagement and long-term follow-up are recommended to maintain donation frequency and ensure blood safety.

## 1 Introduction

Donor reentry is a formal pathway that allows individuals temporarily deferred due to reactive screening results for major transfusion-transmissible pathogens—including hepatitis B virus (HBV), hepatitis C virus (HCV), human immunodeficiency virus (HIV), and *Treponema pallidum* (TP)—to requalify as eligible donors after completing a mandated deferral period and passing supplemental confirmatory testing (1). As a key donor-care and retention initiative, such programs are essential for differentiating true infections from false-positive screening outcomes. This process mitigates the unnecessary and substantial loss of eligible donors, alleviates the documented psychological distress associated with deferral, and contributes to a stable blood supply.

The need for effective reentry protocols is underscored by two major concerns: the psychological impact on donors and the scale of donor loss. Evidence indicates that false-positive test notifications cause psychological distress in up to 86% of affected donors (2). Quantitatively, the loss of donors is significant; in China alone, over 800,000 donations were discarded and donors permanently deferred between 2002 and 2011 based on non-repeatable serological results (3). Similarly, the American Red Cross reported approximately 90,000 first-time donor deferrals from 1995 to 2002 due to unconfirmed reactive results for HBV, HCV, and HIV (4).

While the value of systematic reentry programs is well recognized, their structure and efficacy vary across regions, influenced by local donor demographics and implementation practices. Although several studies have reported donor reentry outcomes in other Chinese cities (5–9), the implementation of reentry protocols remains non-mandatory and varies considerably across regions. A detailed characterization of reentry practices in Chongqing—a major metropolitan area in southwestern China—has not been thoroughly documented. Given the city’s substantial population and persistent need for a stable blood supply, a clear understanding of local reentry outcomes and influencing factors is crucial for refining donor management strategies. Therefore, this study retrospectively analyzed the reentry process among voluntary blood donors in Chongqing from 2022 to 2024, with a specific focus on pathogen-specific requalification rates, the role of educational attainment, and post-reentry donation behavior—factors that have not been comprehensively examined in prior studies. The findings aim to provide evidence-based insights that can guide the optimization of reentry protocols and support the sustainability of blood donation programs in this region.

## 2 Materials and methods

### 2.1 Study subjects

The study subjects were previous voluntary blood donors who had been deferred due to reactive screening results. The inclusion criteria were as follows: during the last donation screening, serological markers for any of the pathogens—hepatitis B virus (HBV), hepatitis C virus (HCV), human immunodeficiency virus (HIV), or *Treponema pallidum* (TP)—showed single-test, single-reagent reactivity, while nucleic acid testing (NAT) results for HBV, HCV, and HIV were non-reactive (for HIV, a negative confirmatory test was required). Donors who expressed a willingness to donate again could actively apply for re-entry after the corresponding deferral period had elapsed (6 months for HBV and HCV; 3 months for HIV and TP). All donors provided informed consent and signed a re-entry application form before blood specimens were collected. This study included 808 donors who actively applied and were approved to enter the re-entry protocol at the Chongqing Blood Center between January 2022 and December 2024.

### 2.2 Specimen processing

From each eligible donor re-entering the blood donation program, two venous blood samples were collected. One sample consisted of 5 mL whole blood anticoagulated with EDTA-K₂, which was processed for serological testing by centrifugation at 1760 × g for 10 min. The other sample consisted of 8 mL whole blood anticoagulated with EDTA-K₂ and containing an inert separation gel, intended for nucleic acid testing (NAT). This tube was centrifuged at 2000 × g for 15 min at 6 °C within 4 h after collection.

### 2.3 Reagents and instruments

The following reagents were used for serological testing, all meeting manufacturers’ specifications. HBsAg kits from DiaSorin (Italy) had 100% sensitivity and 99.97% specificity, and Wantai (Beijing) kits had 100% sensitivity and 100% specificity. Anti-HCV kits from Wantai (Beijing) exhibited 100% sensitivity and 100% specificity, while Lizhu (Zhuhai) kits showed 99.84% positive agreement and 99.93% negative agreement. HIV Ag/Ab kits from Wantai (Beijing) had 100% sensitivity and 100% specificity; Bio-Rad (France) kits demonstrated 100% sensitivity (745/745 confirmed positives) and 99.95% specificity, with p24 antigen sensitivity <25 pg./mL; DiaSorin (UK) kits were used per manufacturer’s specifications. Anti-TP kits from Kehua (Shanghai) met national reference standards, and Wantai (Beijing) kits had 100% sensitivity and 100% specificity. Nucleic acid testing used the Procleix Ultrio Elite assay (Grifols, Spain), with 95% detection limits of 4.3 IU/mL for HBV DNA, 3.0 IU/mL for HCV RNA, and 18.0 IU/mL for HIV RNA.

### 2.4 Supplementary or confirmatory testing

For detection of HBcAb, a chemiluminescence immunoassay kit from Sichuan Wonteng Biotechnology Co., Ltd. (China) was used. According to the manufacturer’s instructions, the assay demonstrated a sensitivity of 99.46% and a specificity of 99.59% in a clinical evaluation involving 1,105 samples. The HIV antigen/antibody confirmatory test was performed by the Jiulongpo District Centers for Disease Control and Prevention (CDC). All tests were conducted on a Cobas e 602 immunoassay analyzer (Roche, USA). All procedures were performed in strict accordance with the manufacturers’ instructions, and all reagents were used within their validity period.

### 2.5 Reentry strategy

Samples from reentry donors were tested for HBsAg, anti-HCV, HIV Ag/Ab, and anti-TP using reagents from two different manufacturers, following standard screening procedures. Individual nucleic acid testing (NAT) was performed for HIV RNA, HCV RNA, and HBV DNA to ensure accurate viral detection. Additionally, a chemiluminescence assay for HBcAb was conducted based on the donor’s prior HBsAg deferral record. Samples showing single-reagent reactivity for HIV Ag/Ab were forwarded to the CDC for confirmatory testing.

### 2.6 Determination of reentry eligibility

**Figure 1:**
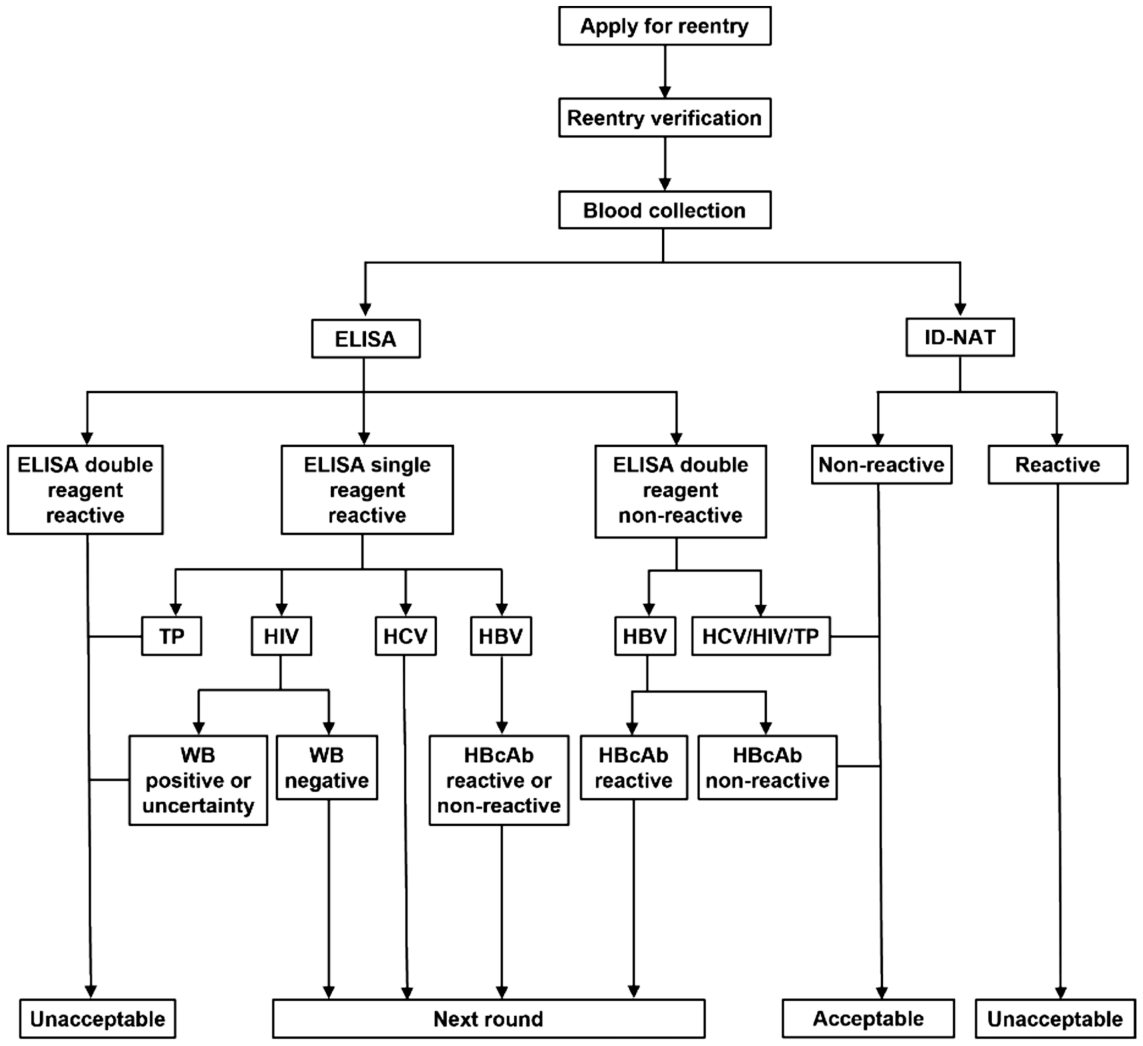
Blood donor reentry strategy. ID-NAT, individual donation nucleic acid testing; WB, western blot.

Reentry eligibility was determined based on testing results and classified into three categories: eligible, ineligible, or follow-up. NR = non-reactive; R = reactive. Eligible donors were allowed to resume voluntary blood donation after a three-month waiting period. The reentry testing algorithm is illustrated in Figure 1.

#### 2.6.1 HBsAg

Eligible: Both ELISA NR + NAT NR + HBcAb-IgG NR.

Ineligible: Both ELISA R and/or NAT R.

Follow-up: NAT NR + (single ELISA R OR HBcAb-IgG R) → retest at 6 months.

#### 2.6.2 Anti-HCV

Eligible: Both ELISA NR + NAT NR.

Ineligible: Both ELISA R and/or NAT R.

Follow-up: NAT NR + single ELISA R → retest at 6 months.

#### 2.6.3 HIV Ag/Ab

Eligible: Both ELISA NR + NAT NR.

Ineligible: Both ELISA R and/or NAT R OR (NAT NR + single ELISA R + CDC positive/indeterminate).

Follow-up: NAT NR + single ELISA R + CDC negative → retest at 3 months.

#### 2.6.4 Anti-TP

Eligible: Both ELISA NR + NAT NR.

Ineligible: Both ELISA R and/or NAT R OR NAT NR + single ELISA R.

### 2.7 Statistical analysis

Statistical analysis was performed using SPSS software (version 27.0). Categorical data were presented as numbers or percentages, and comparisons between groups were conducted using the chi-square test. A *p*-value < 0.05 was considered statistically significant. For the analysis of educational attainment, reentry success was defined as meeting all testing criteria, and the denominator was the total cohort of 808 eligible donors.

## 3 Results

### 3.1 Profile of donors eligible for reentry

A total of 808 blood donors were enrolled in the reentry program. As shown in Table 1, most applicants showed reactivity for HIV markers (*n* = 305, 37.75%), followed by HBV (*n* = 214, 26.49%), HCV (*n* = 174, 21.53%), and TP (*n* = 115, 14.23%). Overall, 489 donors (60.52%) were successfully re-entered, 181 (22.40%) proceeded to the next testing round, and 138 (17.08%) failed reentry. Among the different pathogens, donors reactive for HIV markers had the highest reentry success rate (210/305, 68.85%), while HBV-reactive donors had the lowest (87/214, 40.65%). Notably, nearly half of the donors entering the next testing round were reactive for HBV markers (87/181, 48.07%). The reentry failure rate was highest in the TP-reactive group (35/115, 30.43%), followed by HIV (48/305, 15.74%) and HBV (40/214, 18.69%). The HCV-reactive group showed the highest overall success proportion and the lowest failure rate among the four categories.

**Table 1:** The reentry status of blood donors eligible for reentry.

Pathogen	Apply for reentry (*n*)	Apply for reentry (%)	Successful reentry (*n*)	Successful reentry (%)	Next round (*n*)	Next round (%)	Failed reentry (*n*)	Failed reentry (%)
HBV	214	26.49	87	17.79	87	48.07	40	28.99
HCV	174	21.53	121	24.74	38	20.99	15	10.87
HIV	305	37.75	210	42.94	47	25.97	48	34.78
TP	115	14.23	71	14.52	9	4.97	35	25.36
Total	808		489		181		138	

### 3.2 Characteristics and eligibility rates of reentering donors

Demographic and donation history data of the 808 eligible reentry donors were analyzed and are summarized in Table 2. The cohort included 408 males (50.50%) and 400 females (49.50%), with no significant difference in reentry success rates between sexes (59.56% vs. 60.29%, *p* > 0.05). The largest age group was 31–40 years (30.07%), followed by 21–30 years (26.61%). The distribution of ABO blood types was type O (34.28%), type A (33.79%), type B (23.51%), and type AB (8.42%); no significant association was observed between blood type and reentry outcome (χ^2^ = 1.586, *p* = 0.663). Marital status was largely missing (83.42% unknown) and showed no significant correlation with reentry success (*p* = 0.297). In contrast, education level was significantly associated with reentry outcome (*p* = 0.030): donors with a university degree constituted the largest educational subgroup (43.19%) and achieved a success rate of 53.43%. No statistically significant association was found between occupation and reentry outcome (*p* = 0.287). Regarding donation history, the most common interval between the last donation and reentry application was 3–12 months (36.14%), and most donors had donated only once before (44.93%).

**Table 2:** Characteristics of 808 blood donors eligible for reentry and qualified rates of reentry.

Characteristic	Successful reentry *n*(%)		Next round+Failed reentry *n*(%)		Total *n*(%)		χ^2^	*p*-value
Sex							0.319	0.572
Male	243	59.56	165	40.44	408	50.50		
Female	246	60.29	154	38.50	400	49.50		
Age							70.154	<0.01
≤20	27	6.62	24	47.06	51	6.31		
21 ~ 30	143	35.05	72	33.49	215	26.61		
31 ~ 40	147	36.03	96	39.51	243	30.07		
41 ~ 50	108	26.47	85	44.04	193	23.89		
>50	64	15.69	42	39.62	106	13.12		
ABO blood type							1.586	0.663
A	160	39.22	113	41.39	273	33.79		
B	113	27.70	77	40.53	190	23.51		
O	171	41.91	106	38.27	277	34.28		
AB	45	11.03	23	33.82	68	8.42		
Marital status							2.428	0.297
Married	47	11.52	28	37.33	75	9.28		
Unmarried	41	10.05	18	30.51	59	7.30		
Unknown	401	98.28	273	40.50	674	83.42		
Degree of education							7.003	0.030
Senior high school or below	168	41.18	95	36.12	263	32.55		
University	218	53.43	131	37.54	349	43.19		
Unknown	103	25.25	93	47.45	196	24.26		
Occupation							7.381	0.287
Student	93	22.79	60	39.22	153	18.94		
Freelancer	38	9.31	21	35.59	59	7.30		
Office worker	38	9.31	23	37.70	61	7.55		
Worker	32	7.84	25	43.86	57	7.05		
Civil worker /teacher/healthcare worker/Military personnel	50	12.25	22	30.56	72	8.91		
Farmer	25	6.13	9	26.47	34	4.21		
Others	213	52.21	159	42.74	372	46.04		
Interval (Month)							4.297	0.367
3 ~ 12	188	46.08	104	35.62	292	36.14		
13 ~ 24	77	18.87	65	45.77	142	17.57		
25 ~ 36	39	9.56	26	40.00	65	8.04		
37 ~ 48	28	6.86	20	41.67	48	5.94		
>49	157	38.48	104	39.85	261	32.30		
Number of blood donations							22.344	<0.01
1	194	47.55	169	46.56	363	44.93		
2	76	18.63	53	41.09	129	15.97		
3 ~ 5	130	31.86	61	31.94	191	23.64		
6 ~ 10	39	9.56	22	36.07	61	7.55		
11 ~ 20	25	6.13	10	28.57	35	4.33		
>20	25	6.13	4	13.79	29	3.59		

### 3.3 Factors associated with progression to the next testing round or reentry failure

**Figure 2:**
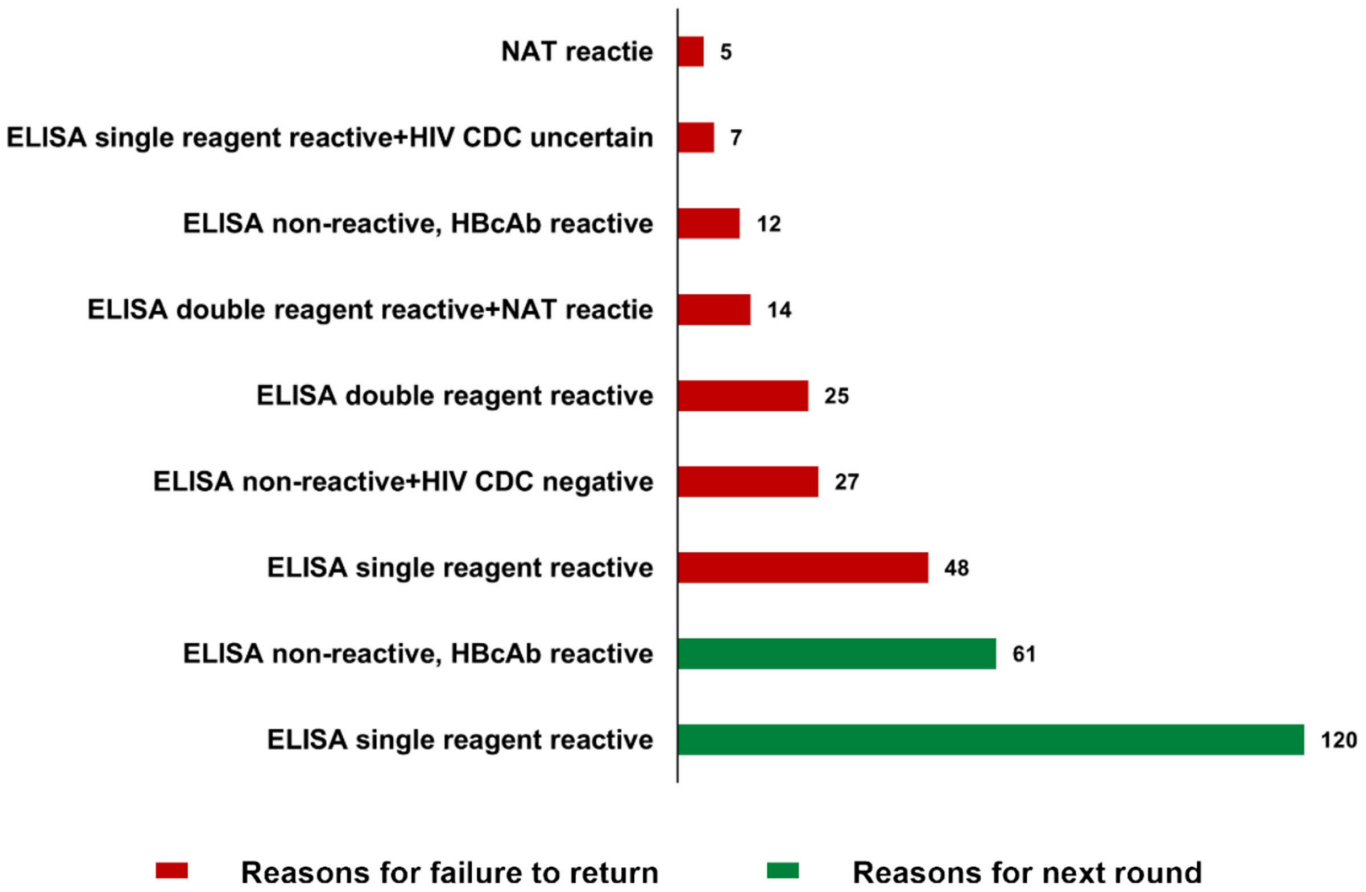
Reasons for the next round and failure.

Reasons for donors either advancing to the next testing round or failing reentry are presented in Figure 2. The most frequent reason was single-reagent ELISA reactivity (*n* = 61), followed by non-reactive ELISA with reactive HBeAb (*n* = 48). Other contributing factors included non-reactive ELISA combined with a negative HIV confirmatory test by CDC (*n* = 25), double-reagent ELISA reactivity (*n* = 14), and double-reagent ELISA reactivity together with reactive NAT results (*n* = 12).

### 3.4 Blood donation volume contributed by successfully re-entered donors

A total of 489 donors successfully qualified for reentry and became eligible to donate after the three-month waiting period. Of these, 240 donors (49.08%) returned to make at least one donation during the follow-up period, contributing to the blood collections detailed in Table 3. The remaining 249 donors had not yet returned by the end of the study period. For whole blood donations, 343 collections were performed, predominantly at a volume of 400 mL (*n* = 234, 68.22%), resulting in a total whole blood collection of 119,000 mL. For apheresis platelet donations, 293 procedures were carried out, with double-unit collections (2 U) being the most common (*n* = 207, 70.65%). The total apheresis platelet yield was 500 U.

**Table 3:** Blood donation volume of reentry donors.

Component	Whole blood (mL)				Apheresis platelets (U)		
Donation volume	200	300	400	Total	1	2	Total
Number of blood donations	73	36	234	343	86	207	293
Blood collection volume	14,600	10,800	93,600	119,000	86	414	500

### 3.5 Donation frequency among returning re-entered donors

Among the 240 donors who returned for subsequent donations, the majority donated once (*n* = 127, 52.92%), followed by those who donated twice (*n* = 56, 23.33%). A smaller proportion donated 3–10 times (*n* = 46, 19.17%), and only 11 donors (4.58%) contributed more than 10 times after reentry (Table 4).

**Table 4:** Re-donation analysis of 240 successfully re-entered donors.

Number of re-donations	*n*	Rate (%)
1	127	52.92
2	56	23.33
3 ~ 10	46	19.17
>10	11	4.58
Total	240	

### 3.6 Reasons for disqualification in subsequent donations after reentry

Among the donors who successfully re-entered, 33 (13.75% of 240) were subsequently disqualified from further donation. The reasons for disqualification are summarized in Table 5. Reactivity for HIV markers was the most common cause, accounting for 16 cases (48.48%), followed by HBV (*n* = 7, 21.21%) and TP (*n* = 5, 15.15%). Other reasons included elevated ALT levels (*n* = 4, 12.12%) and reactive TRI-NAT results (*n* = 1, 3.03%). Notably, no donor was disqualified due to HCV reactivity during the post-reentry follow-up.

**Table 5:** Distribution of 33 unqualified blood donors after reentry.

Reasons	*n*	Rate (%)
HBV	7	21.21
HCV	0	0.00
HIV	16	48.48
TP	5	15.15
ALT	4	12.12
TRI-NAT	1	3.03
Total	33	

TRI-NAT, triplex nucleic acid testing for simultaneous detection of HBV, HCV, and HIV.

## 4 Discussion

False-reactive results remain a recognized challenge in blood-donor screening using ELISA, arising from factors such as reagent characteristics, cut-off (gray-zone) settings, laboratory conditions, and specimen quality. Among these, the deliberate use of gray-zone cut-offs to enhance safety contributes substantially to false-reactivity rates. Studies report that gray-zone samples yield false-positive rates as high as 63.64% for HBsAg and 96.08% for anti-HCV (10). Similarly, false-positive rates for HIV antigen/antibody testing have been reported to reach 82% (11). Indiscriminate deferral of donors based on such results not only diminishes donor satisfaction but also leads to avoidable loss of blood resources. Reentry programs offer a systematic approach to mitigate these issues. In China, the “Guideline for Reentry of Reactive Blood Donors in Blood Screening (T/CSBT 002–2019)” was issued in 2019 to standardize practice. However, as this guideline is non-mandatory and does not address reentry for donors with reactive nucleic-acid screening results, implementation varies regionally. Currently, only a few provinces such as Zhejiang and Jiangsu have adopted unified provincial reentry protocols.

Against this backdrop, our study retrospectively analyzed 808 voluntary blood donors who applied for reentry at the Chongqing Blood Center between 2022 and 2024. The overall reentry success rate was 60.52%, which falls within the mid-range reported in other Chinese cities—from 56.62% in Shaoxing (5) and 58.74% in Hangzhou (6) to 74.85% in Nanning (7) and 83.01% in Shenzhen (9). Such regional variation likely reflects differences in donor demographics, screening algorithms, deferral policies, and donor-recruitment approaches. Notably, our observed rate substantially exceeds the 2.7% active reentry rate reported in Changsha (8), where reentry relied solely on donor self-initiation without proactive recall, underscoring the impact of program design on donor participation.

Internationally, reentry guidelines vary in stringency. The WHO recommends standardized protocols to balance safety and donor retention (12). In the US, the American Red Cross permits reentry under specific conditions, though deferral registries have shown limited effectiveness (4). Canada’s program (since 2010) allows requalification via a different testing platform; long-term follow-up showed an 8.4% second-deferral rate over 3 years, significantly lower when a different platform was used (1.8% vs. 21.4%, *p* < 0.0001) (13). In Dalian, China, 61.9% of deferred donors with unconfirmed results were confirmed false-positive, supporting reentry feasibility (3). However, HBV complexity (e.g., occult infection) requires careful marker interpretation. China’s 3-month waiting period with dual-ELISA plus NAT balances safety and accessibility, though further validation of residual risk is needed (14). These international data collectively support refining reentry programs.

Reentry success varied significantly by pathogen type. Donors with initial HIV marker reactivity achieved the highest requalification rate (68.85%), consistent with the high frequency of false-positive single-reagent results in low-prevalence populations (15). In contrast, HBV-reactive donors had the lowest requalification rate (40.65%), which was lower than the 55.05% reported in Anhui (16). This disparity may relate to the complex serology of HBV infection, including occult infection or resolved infection with persistent anti-HBc reactivity (17–21).

Among demographic factors, only higher educational attainment was significantly associated with reentry success, aligning with reports from Guangzhou and other regions where better-educated donors showed greater understanding of false-reactive results and stronger motivation to regain eligibility (22). Sex, ABO blood type, marital status, and occupation were not significantly correlated with success in our cohort—a finding that diverges from some earlier studies identifying male sex and repeat-donation history as positive predictors, possibly reflecting regional donor characteristics or methodological differences.

Post-reentry donation behavior further illustrated the program’s impact. Of the 489 successfully re-entered donors, 240 (49.08%) returned to donate at least once, contributing substantially to blood collections. However, most donated only once or twice thereafter, suggesting that the initial reactive result may have a lingering effect on long-term commitment. This highlights the need for continued donor communication and support even after successful reentry. Moreover, 13.75% (33/240) of these donors were disqualified in subsequent donations—a rate lower than that in Hangzhou (18.42%) (6) but higher than that in Wuhan (11.27%) (23). Reactivity for HIV, HBV, or TP remained the primary reasons for disqualification, emphasizing that reentry does not eliminate future risk and that ongoing vigilant screening remains essential.

Compared to previous studies that primarily reported overall reentry rates (5–9), our study extends the literature by systematically analyzing the association between educational level and reentry success, providing detailed data on post-reentry donation frequency and disqualification reasons, and evaluating pathogen-specific requalification outcomes. These additional dimensions offer practical insights for optimizing reentry protocols in diverse regional contexts.

To contextualize the post-reentry donation behavior observed in our cohort, we examined the general blood donation landscape in Chongqing. According to data published by the Chongqing Municipal Health Commission, the city achieved a blood donation rate of 11.3 per 1,000 population in 2022, with a total of 362,500 donations (24). Within this broader context, the 49.08% return rate among our successfully re-entered donors is notable. It suggests that while the reentry process effectively reintegrates a substantial proportion of deferred donors into the eligible pool, sustaining their long-term engagement remains a challenge, as most returned only once or twice. This finding underscores the need for targeted follow-up and support strategies specifically for re-entered donors to maximize their contribution to the overall blood supply.

Several limitations should be acknowledged. First, as a single-center study, the findings may not be fully generalizable to other regions with different donor profiles and screening practices. Second, although the sample size of 808 donors is comparable to similar studies (5–9), it remains relatively modest for drawing definitive conclusions, particularly for subgroup analyses. Multi-center studies with larger cohorts are needed to validate and extend our findings. Third, the follow-up period (2022–2024) was relatively short (approximately 2 years), which may not capture long-term donor retention patterns or rare adverse events. Extended observation over 3–5 years would better clarify sustained donor engagement and the long-term safety of reentry programs. Fourth, we did not collect qualitative data on donor motivation or reasons for non-participation, limiting insight into barriers to reentry. Finally, although our protocol adhered to national guidelines, it represents one of several possible approaches; comparative studies of alternative algorithms would be valuable. Additionally, while the reentry algorithm in this study followed the sequential testing pathway recommended by the Chinese Blood Transfusion Association guidelines (T/CSBT 002–2019), the study was not designed to compare the performance of different testing methods. Future prospective studies incorporating formal inter-method agreement analyses, such as Cohen’s kappa statistics, would be valuable to further validate the consistency and robustness of reentry testing protocols across different settings. From a health economics perspective, reentry programs also carry financial implications. The costs of confirmatory testing and donor communication must be weighed against the benefits of retaining experienced donors and avoiding new donor recruitment. With 489 donors successfully re-entered and 240 returning to donate, the program likely yields net savings; however, formal cost-effectiveness analyses are warranted in future research to guide resource allocation.

## 5 Conclusion

This study confirms that a systematically implemented reentry process can effectively reintegrate a substantial proportion of single-reagent-reactive donors into the eligible donor pool in Chongqing, thereby reducing unnecessary donor loss and supporting blood supply sustainability. Reentry success is influenced by pathogen type and donor education level, highlighting the need for tailored communication and risk-stratified testing algorithms. To further optimize reentry practice, we recommend: (1) strengthening the promotion and accessibility of the reentry policy, especially toward first-time and less-educated donors, to ensure broad awareness and participation; (2) implementing pathogen-specific algorithms, such as anti-HBc testing for HBV-reactive donors, to better balance safety and donor return; and (3) establishing proactive follow-up and supportive communication after reentry to promote sustained donation commitment. Continued research comparing reentry protocols and assessing long-term safety will help refine and strengthen these programs nationwide, ensuring their effectiveness in balancing donor care with blood safety.

## Data Availability

The raw data supporting the conclusions of this article will be made available by the authors, without undue reservation.

## References

[1] Li ZY. Interpretation of the guideline for reentry of reactive blood donors in blood screening testing. Health Everyone. (2022) 6:36–7. doi: 10.20252/j.cnki.rrjk.2022.06.013

[2] Delage G Myhal G Grégoire Y Simmons-Coley G. Donors' psychological reactions to deferral following false-positive screening test results. Vox Sang. (2014) 107:132–9. doi: 10.1111/vox.12143, 24646091

[3] Deng X Zang L Wang X Chen H Liu J Gao Y . Follow-up program for blood donors with unconfirmed screening results reveals a high false-positive rate in Dalian, China. Transfusion. (2020) 60:334–42. doi: 10.1111/trf.15656, 31909495

[4] Cable R Musavi F Notari E Zou S. Limited effectiveness of donor deferral registries for transfusion-transmitted disease markers. Transfusion. (2008) 48:34–42. doi: 10.1111/j.1537-2995.2007.01480.x, 17894796

[5] Zhu S Sun J Lu Y. Perspectives on the re-engagement of screen-reactive blood donors. Beijing Med J. (2024) 46:873–7. doi: 10.15932/j.0253-9713.2024.10.015

[6] Lu Y Ding W Guo WY Zhu FM Zhang J. Analysis of the reentry status of blood donors with reactive bloodborne pathogen screening markers in Hangzhou City. Chinese J Prevent Med. (2023) 57:1565–70. doi: 10.3760/cma.j.cn112150-20221208-01187, 37859372

[7] Li X Jiang Y Li Y Wei Z Zhu Q Zhang L . Analysis of requalification test results and subsequent donation status in 676 blood donors in Nanning City. Lab Med Clin Med. (2021) 18:3185–7. doi: 10.3969/j.issn.1672-9455.2021.21.032

[8] Li S Kang T Yuan Y Luo J. Reentry of reactive blood donors in Changsha area: a retrospective analysis. Chin J Blood Transfus. (2024) 37:444–8. doi: 10.13303/j.cjbt.issn.1004-549x.2024.04.012

[9] Zheng X Xu X Zeng J Chen Y Liu H Xiong W. Analysis of the returning test results of HBV and HCV reactive in volunteer blood donors in Shenzhen city and discussion on the strategy. Chin J Blood Transfus. (2019) 32:1024–7. doi: 10.13303/j.cjbt.issn.1004-549x.2019.10.014

[10] Song M Ren F Gong X Wang Z. False-positive analysis of HBsAg and anti-HCV ELISA screening discordant samples in blood donors. Beijing Med J. (2013) 35:391–5. doi: 10.15932/j.0253-9713.2013.05.004

[11] Li L Xu T Yang T Zang L Cheng W Lin H . Establishing a reentry procedure for human immunodeficiency virus screening-reactive donors in China. Transfusion. (2016) 56:195–202. doi: 10.1111/trf.13282, 26360920

[12] World Health Organization. Blood Donor Selection: Guidelines on Assessing Donor Suitability for Blood Donation. Geneva: World Health Organization (2012). 23700651

[13] Grégoire Y Germain M Delage G. Factors associated with a second deferral among donors eligible for re-entry after a false-positive screening test for syphilis, HCV, HBV and HIV. Vox Sang. (2018) 113:339–44. doi: 10.1111/vox.12644, 29508402

[14] Deng X Zang L Candotti D. Re-entry evaluation of Chinese blood donors with unconfirmed hepatitis B screening results. Viruses. (2022) 14:2545. doi: 10.3390/v14112545, 36423154 PMC9698129

[15] Buccheri R Warden DE Oikawa M Grebe E Miranda C Amorim L . Assessing HIV trends among blood donors in five Brazilian blood centers: the impact of individual donor assessment. Transfusion. (2025) 65:685–95. doi: 10.1111/trf.18168, 39995013 PMC12005579

[16] Jiang F Lyu R Zhao Y Li S Wang C Liu Z . Analysis on reentry situation of HBsAg single reagent reactive blood donors in Anhui Province. J Exp Hematol. (2020) 28:1391–6. doi: 10.19746/j.cnki.issn.1009-2137.2020.04.053 32798432

[17] Hu W Jiang N Zhu S Lin H. Study on reentry evaluation mode for blood donors used to be HBV reactive in Jiangsu Province. J Exp Hematol. (2022) 30:264–9. doi: 10.19746/j.cnki.issn.1009-2137.2022.01.044 35123638

[18] van de Laar TJ Hogema BM Molenaar-De Backer MW Marijt-Van Der Kreek T Zaaijer HL. Blood donor screening in the Netherlands: universal anti-HBc screening in combination with HBV nucleic acid amplification testing may allow discontinuation of hepatitis B virus antigen testing. Transfusion. (2021) 61:2116–24. doi: 10.1111/trf.16420, 33899233

[19] Takuissu GR Kenmoe S Amougou Atsama M Atenguena Okobalemba E Mbaga DS Ebogo-Belobo JT . Global epidemiology of occult hepatitis B virus infections in blood donors, a systematic review and meta-analysis. PLoS One. (2022) 17:e0272920. doi: 10.1371/journal.pone.0272920, 35994469 PMC9394819

[20] Harvala H Reynolds C Gibney Z Derrick J Ijaz S Davison KL . Hepatitis B infections among blood donors in England between 2009 and 2018: is an occult hepatitis B infection a risk for blood safety? Transfusion. (2021) 61:2402–13. doi: 10.1111/trf.16543, 34114670

[21] Makokha GN Zhang P Hayes CN Songok E Chayama K. The burden of hepatitis B virus infection in Kenya: a systematic review and meta-analysis. Front Public Health. (2023) 11:986020. doi: 10.3389/fpubh.2023.986020, 36778557 PMC9909240

[22] Ling M Liang J Ma W Lao L Xie J. Analysis of the return of reactive blood donors to blood screening in Panyu District, Guangzhou City. China Mod Med. (2025) 32:94–8. doi: 10.3969/j.issn.1674-4721.2025.18.20

[23] Xu T Yu Q Ke S Cai Y Xie S Xiong J . Study on reentry strategy and results of blood donors with single reagent reactivity in Wuhan area. J Exp Hematol. (2025) 33:530–7. doi: 10.19746/j.cnki.issn.1009-2137.2025.02.033, 40326130

[24] Chongqing Municipal Health Commission. (2023). Event Report on the 20th World Blood Donor Day [Internet]. Chongqing: CMHC. Available online at: http://www.wsjkw.cq.gov.cn/ztzl_242/jrjnrhdzt/sjxxzr/202306/t20230610_12052650.html (Accessed February 28, 2026).

